# Single-Cell BCR and Transcriptome Analysis Revealed Potent Neutralizing Antibodies in Response to HAdV-4 Infection

**DOI:** 10.3390/ijms27208952

**Published:** 2026-10-09

**Authors:** Tingting Yu, Xiaolei Ye, Zheng Zhu, Wanrong Zhang, You Yang, Jianrong Wang, Peng Lv, Xiangyang Chi, Guanying Zhang, Zhengshan Chen, Yunzhu Dong

**Affiliations:** National Key Laboratory of Advanced Biotechnology, Academy of Military Medical Sciences, Beijing 100071, China

**Keywords:** single-cell sequencing, human adenovirus serotype 4, neutralizing monoclonal antibody

## Abstract

Human adenovirus type 4 (HAdV-4) is a predominant serotype causing acute respiratory infections, for which no antibody drugs have been clinically approved to date, resulting in an urgent unmet medical need. This study aimed to identify highly potent neutralizing antibodies by dissecting the B cell immune response after infection, thereby laying a foundation for vaccine design and antiviral therapy. Peripheral blood mononuclear cells (PBMCs) were isolated from a convalescent patient who exhibited the most robust neutralizing antibody titers among 16 HAdV-4 convalescent volunteers. A total of 2710 B cells were isolated from the peripheral blood of this patient, and the characteristics of B cell subsets and B cell receptor (BCR) repertoires were comprehensively analyzed by integrating scRNA-seq and scVDJ-seq technologies. Candidate antibodies were subsequently selected for a series of functional and mechanistic validations based on the characteristics of BCRs and origination. Transcriptomic profiling classified memory B cells into three functionally heterogeneous subpopulations designated as MB sub1, sub2 and sub3. Combined with the characteristics of BCR, 92 selected antibodies from sub2 and sub3 were synthesized for further functional validation. Three potent neutralizing antibodies were successfully identified, among which 1QH7 demonstrated potentially potent virus neutralization capability in vitro and conferred robust in vivo protection in the *Stat1*-deficient mouse model. We further employed AlphaFold 3 to predict the complex structure of 1QH7 with the HAdV-4 Hexon protein and validated the key binding residues through site-directed mutagenesis. Additionally, the study elucidated the viral neutralization mechanism mediated by TRIM21. We established an efficient antibody screening strategy based on integration of scRNA-seq and scVDJ-seq and isolated a potent neutralizing antibody, 1QH7. This study lays a solid foundation for the rapid pathogen-bait-free, cell subset-guided screening antibody route for novel emerging infectious disease outbreaks.

## 1. Introduction

Human adenovirus (HAdV) is a globally widespread pathogen, and human adenovirus type 4 (HAdV-4) is a common serotype causing acute respiratory infections [1,2,3]. HAdV-4 infection occurs more frequently in infants, young children, and immunocompromised individuals, with some cases progressing to severe pneumonia and even death [4]. While HAdV-4 infections were previously often associated with outbreaks in collective settings such as military camps, their detection rate in the general community has increased significantly in recent years [5]. A retrospective study from Guangzhou Children’s Hospital revealed that among lung tissue samples from children who died of pneumonia between 1988 and 2005, HAdV-4 accounted for as many as 75% of all adenovirus-positive cases, suggesting it may be a critical serotype in fatal pediatric pneumonia [6,7]. However, there is currently a lack of specific antiviral drugs targeting HAdV-4 and vaccines licensed for the general population, making supportive care the mainstay of treatment [8,9]. Therefore, developing efficient and safe specific antiviral strategies represents an urgent and unmet clinical need.

Currently, antibody-based therapy has emerged as a highly promising interventional strategy against viral infectious diseases. Human monoclonal antibodies targeting key viral surface proteins, such as the spike protein or envelope protein, have increasingly demonstrated their therapeutic and prophylactic efficacy against various emerging and re-emerging infectious diseases, including SARS-CoV-2 [10], human respiratory syncytial virus [11], and Ebola virus [12]. The safety and clinical efficacy of these antibodies have been confirmed in multiple randomized controlled trials, showing an ability to effectively reduce viral load and decrease the risk of severe disease and death [13,14,15]. However, traditional methods for screening neutralizing antibodies suffer from low throughput and are time-consuming. They rely on the individual validation of a large number of clones, a process that typically takes several months [16]. This approach struggles to facilitate a rapid response in the face of novel or sudden infectious disease outbreaks. Therefore, establishing new, efficient, and rapid pathways for antibody discovery has become an urgent necessity.

Breakthrough advances in single-cell sequencing technology have provided a new solution for efficient antibody screening. The high-throughput single-cell BCR sequencing workflow established by Leonard D. Goldstein’s team, based on the 10x Genomics platform, has fully demonstrated the potential of this technology. It not only recovers 81% of known anti-chicken ovalbumin (OVA) lineages but also discovers 710 candidate lineages missed by traditional methods, with 99% demonstrating specific binding. This highlights its advantage in identifying rare antibodies [17]. Leveraging microfluidic platforms, single-cell sequencing technology enables the high-throughput parallel capture of transcriptomes and VDJ sequences from tens of thousands of individual B cells, achieving automated BCR pairing [18]. This approach not only shortens the antibody discovery timeline from months to days but, when integrated with bioinformatic in-depth mining of B cell repertoire diversity, provides a powerful tool for rapidly pinpointing candidate antibodies with specific expression features or mutation patterns.

In this study, we established an integrated antibody discovery strategy combining single-cell sequencing with functional validation. We isolated PBMCs from the HAdV-4 convalescent donor with the highest neutralizing antibody titers among 16 volunteers. From this donor, 2710 B cells were sorted for simultaneous scRNA-seq and scVDJ-seq. Integrated analysis of the transcriptome and BCR repertoire precisely classified the memory B cells into three functionally heterogeneous subsets (MB sub1, sub2, and sub3). Adaptive Immune Receptor Repertoire Analysis revealed that MB sub2 and sub3 exhibited distinctive features in terms of somatic hypermutation (SHM) levels and HCDR3 clone diversity, suggesting their role as the primary source of antigen-specific and affinity-matured antibodies. Based on this analysis, we targeted MB sub2 and MB sub3, synthesizing 92 antibody genes for expression and validation according to their enriched specific V-J gene combinations. Among these, antibody 1QH7 demonstrated potent neutralizing activity in vitro and showed significant prophylactic and therapeutic efficacy in a *Stat1*-deficient mouse model. This work not only provides a promising therapeutic antibody candidate against HAdV-4 but also validates the efficiency and applicability of the single-cell sequencing-based antibody discovery platform in responding to emerging infectious diseases.

## 2. Results

### 2.1. Single-Cell Transcriptomic Profiling Reveals B Cell Heterogeneity Landscape

**Figure 1:**
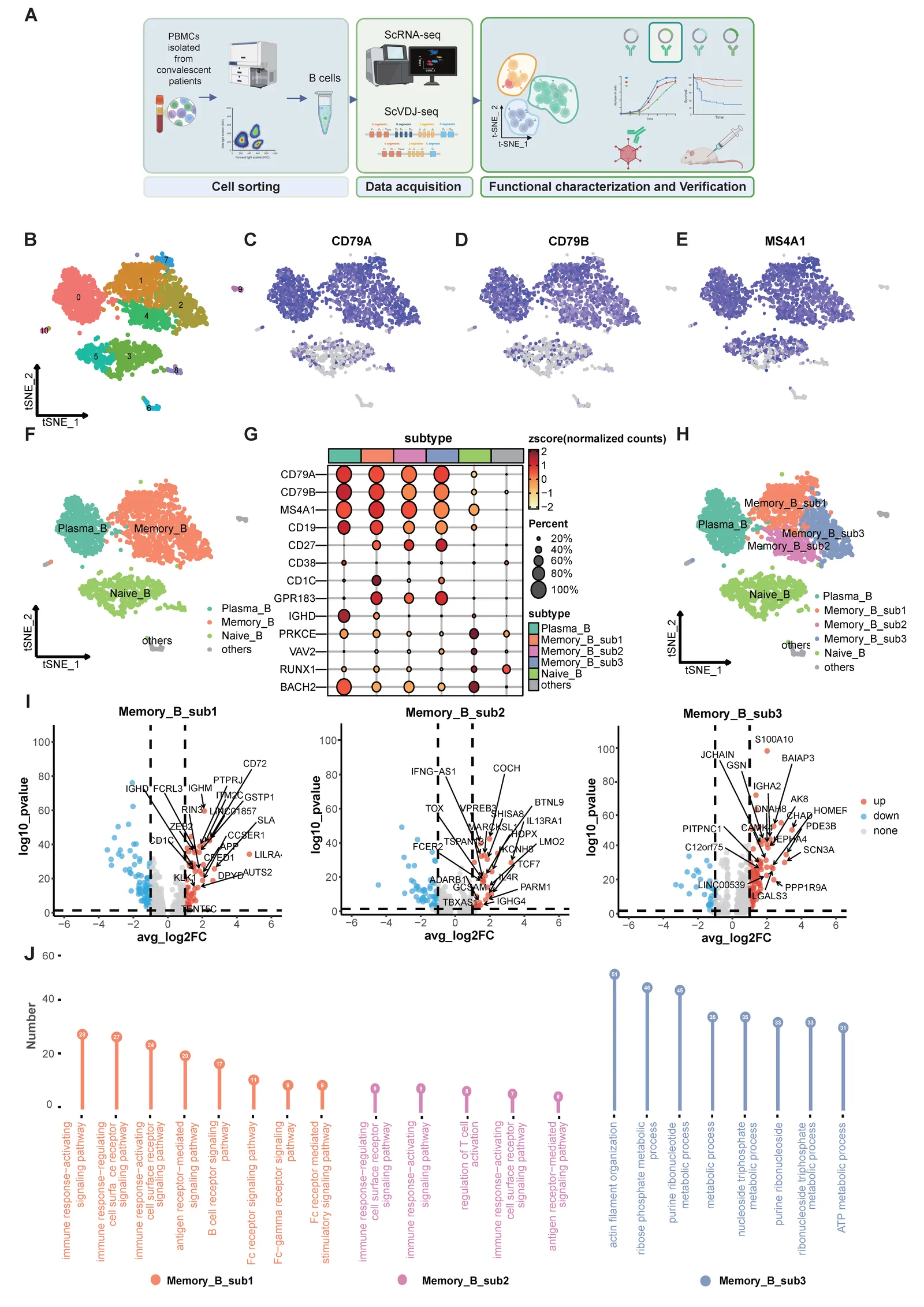
The schematic overview research design and analysis of single-cell RNA sequencing. (**A**) The experimental workflow of the potential neutralizing antibody identification from convalescent patients, including cell sorting, ScRNA-seq, ScVDJ-seq, functional characterization and verification. (**B**) Principal component analysis (PCA) and t-distributed stochastic neighbor embedding (tSNE) separates cells into 10 clusters by gene expression profile. (**C**) The distribution and expression of B cell marker gene *CD79A*. (**D**) The distribution and expression of B cell marker gene *CD79B*. (**E**) The distribution and expression of B cell marker gene *MS4A1*. (**F**) Identification of the main cell population based on gene expression. (**G**) Dot plot showing the marker genes for identification of B cell subpopulations. (**H**) Identification of sub-cell population based on gene expression, including Memory_B_sub1, Memory_B_sub2 and Memory_B_sub3. (**I**) Gene expression differences in three memory B subpopulations; the gene names of the top 20 upregulated expressions were noted. (**J**) GO enrichment analysis based on upregulated genes in three memory B subpopulations.

In contrast to the conventional antibody screening process, analysis of scVDJ-seq and scRNA-seq data enables rapid identification of specific B cell clusters and candidate neutralizing antibodies. High-affinity binding and neutralizing antibodies are more likely to originate from convalescent patients, driven by B cell clonal selection and expansion after virus infection. To facilitate effective candidate antibody screening, peripheral blood mononuclear cells (PBMCs) were isolated from the HAdV-4 convalescent volunteer exhibiting the highest neutralizing antibody titers among 16 donors. Next, scRNA-seq (10× Genomics) and scVDJ-seq were performed successfully on 2710 B cells from a sample which was sorted by flow cytometry (Figure 1A). After the data filtering, unsupervised clustering, cell subset classification and cell type annotation of the Seurat object, 11 cell clusters were identified and plotted in a t-distributed stochastic neighbor embedding (t-SNE) space (Figure 1B). B cells were distinguished by several markers, including *CD19*, *MS4A1*, *CD79A* and *CD79B* [19,20,21], and were highly expressed in total cells, indicating the accurate flow sorting (Figure 1C–E). *GPR138*, *CD1C* [22] and *CD27* [23] were mainly expressed in memory B cells, while *CD38* [23,24] were mainly expressed in Plasma B cells. With reference to the aforementioned characteristic genes, the clusters were manually annotated as naive B cells, plasma cells, memory B cells, and others based on specific expression profiles, which accounted for 25.35%, 23.28%, 47.08% and 4.28% of total cells respectively (Figure 1F). Memory B cells were the primary target population for antibody identification and screening, due to their long lifespan and robust expression of highly specific, affinity-matured BCRs. The difference in B cell marker gene expression, such as *CD1C*, among three subgroups indicated potential functional capabilities among the various memory B subpopulations (Figure 1G). The memory B cell population was divided into three transcriptional distinct subclusters, designated memory B subtype 1 (MB sub1), memory B subtype 2 (MB sub2), and memory B subtype 3 (MB sub3), accounting for 565 cells (44.28%), 287 cells (22.49%), and 424 cells (33.23%), respectively (Figure 1H).

To further characterize the transcriptome of memory B cell subtypes, Differentially expressed genes (DEGs) were identified in three MB subgroups. Gene expression patterns were quantified relative to the other MB populations, with the top 20 upregulated genes annotated (Figure 1I). First, the *CD72* was elevated in MB sub1, which indicated that sub 1 might be the resting memory B cell group. Next, the different gene expression involved in antibody production, including *IGHM*, *IGHD*, *IGHG4* and *IGHA2*, implied that three memory B subgroups express a diverse repertoire of antibody molecules. Gene ontology (GO) analyses were subsequently conducted to gain insight into the functions of different memory B cell subgroups (Figure 1J). As expected, genes in the “immune response-activating signaling pathway” and “B cell receptor signaling pathway” groups were enriched MB sub1, implying antigen identification and memory B cell activation. A similar GO biological process was enriched in MB sub2, but the gene count was lower than that of MB sub1. Unlike MB sub1 and sub2, MB sub3 showed significant enrichment mainly in other aspects, including “actin filament organization”, the “ribose phosphate metabolic process” and the “ATP metabolic process”. This result suggested various functions among three memory B subgroups. We hypothesized that MB sub1 likely functioned primarily in immune surveillance. MB sub2 represented an intermediate or transitional state and might have played a role in immune regulation. In contrast, MB sub3 was primed to differentiate into plasma cells and mainly engaged in preparation for antibody secretion, poised to rapidly produce high-affinity antibodies upon future re-stimulation.

**Figure 2:**
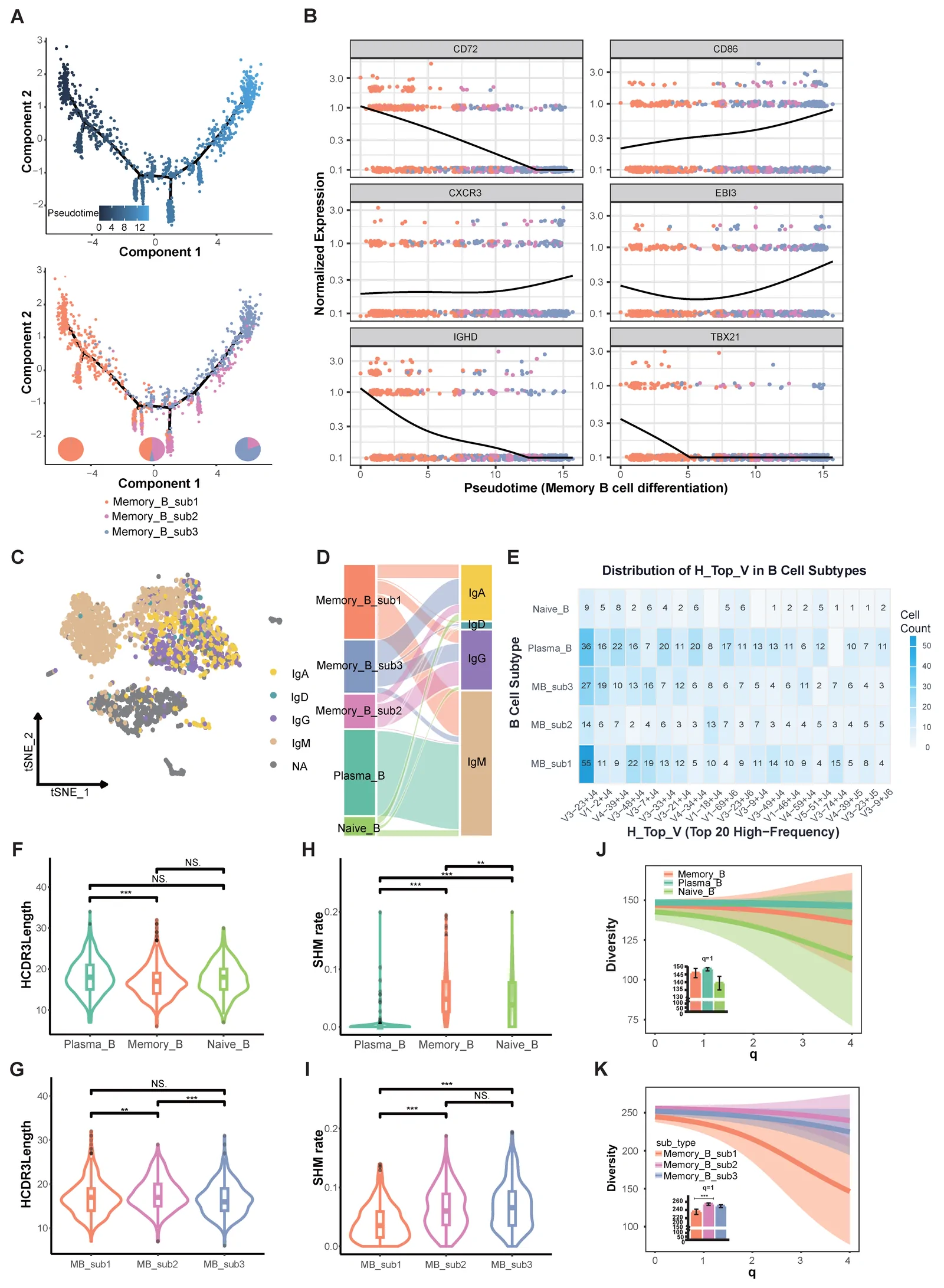
Gene features and BCR V(D)J gene usage in single B cells. (**A**) Transcriptomic-based inference of developmental trajectory for three memory B cell subpopulations with monocle2. Both pseudotime prediction (**upper**) and memory B cell subpopulation distribution (**below**) are shown along the trajectory. (**B**) Pseudotime projection of transcriptional changes in key genes during differentiation along the trajectory. (**C**) The tSNE distribution of various BCR isotypes. (**D**) The proportion of various BCR isotypes originating from B cell subpopulations. (**E**) The frequency of the top 20 frequent combinations of V gene and J gene usage for heavy chains among total cells. (**F**) The difference in the CDR3 length in heavy chains among the main B cell populations. (**G**) The difference in the CDR3 length in heavy chains among memory B cell subpopulations. (**H**) The difference in BCR SHM rate among main B cell populations. (**I**) The difference in BCR SHM rate among memory B cell subpopulations. (**J**) The diversity of BCR among main B cell populations; Shannon index shown below (q = 1). (**K**) The diversity of BCR memory B cell subpopulations; Shannon index shown below (q = 1). NS (not significant), ** *p* < 0.01, *** *p* < 0.001.

To clarify the functional capabilities among the three memory B cell subgroups, trajectory inference analysis was performed using Monocle2. The result showed that MB sub1 was distributed in the initial branching segments while sub2 and sub3 were mainly located in the terminal branching segments of the pseudotime trajectory. The proportions of three memory B cell subpopulations are as presented below at different pseudotime points, and the fractions of MB sub2 and MB sub3 exhibited a gradual increase along the pseudotime trajectory. MB sub2 was found to have an independent population that contained cells in a transitional state towards the other two subpopulations, suggesting a gap between the two major branches (Figure 2A). Further analysis revealed that genes associated with memory B cell activation exhibited dynamic changes along the pseudotime trajectory. The decrease in *CD72*, which is an inhibitory co-receptor of the B cell receptor (BCR), indicates the activation of memory B cells. The activation marker *CD86* demonstrated a gradually increasing expression among memory B cell populations along the trajectory. Similar to the findings of a previous study, *CXCR3* and *EBI3* expression, which were exclusively expressed in activated memory B cells [23], also showed a gradually increasing trend. The expression of *TBX21*, which is vital for effector memory B cell populations and is helpful for rapidly secreting antibody [25], was mainly higher in MB sub1 and decreased along the pseudotime trajectory (Figure 2B). The above results indicate the functional differences among these three groups of memory B cells. MB sub3 may be the main cell population responsible for stabilizing the expression of specific BCRs on the cell surface after activation.

### 2.2. Characteristics of BCR Immune Repertoire in Different B Cell Populations

BCR immune repertoires serve as crucial indicators of B cell function. To evaluate the diversity of BCR repertoires at the single-cell level, we performed scVDJ-seq on the identical sorted B cells. In total, 1972 full-length paired BCRs were identified, accounting for 72.77% of the total B cell population. The expression patterns of BCR antibody isotypes differed across distinct B cell populations (Figure 2C). In total, 21.40% of pre-B cells, 93.03% of memory B cells and 99.21% of plasma cells expressed BCRs. Plasma cells predominantly expressed IGHM (97.62%), whereas memory B cells simultaneously expressed IGHA (27.74%), IGHM (30.25%) and IGHG (31.66%). Three memory B cell subpopulations also exhibited distinct antibody isotype expression profiles. MB sub1 mainly expressed IGHM while MB sub2 and MB sub3 mainly expressed IGHG. Notably, the IGHG derived from MB sub1 and sub2 accounted for 70.02% of total IGHG, implying that potent high-affinity antibodies were likely distributed in MB sub1 and sub2 due to a strong response to viral stimuli [26] (Figure 2D, Supplemental Table S1).

The breadth, magnitude, and clonal diversity of the BCR are critical determinants of effective antibody generation against viral infection. Further analysis of BCR germline gene rearrangement and somatic hypermutation (SHM) characteristics was performed. Detailed variable (V) and joining (J) germline gene usage for heavy chains and light chains is shown in Supplemental Figures S1 and S2. Complementarity-determining region 3 (CDR3), the diversity of which is closely linked to V and joining J gene usage in BCR heavy chains, is widely considered the primary protein loop that determines antibody specificity. The frequency of V-J pairs of heavy chains was calculated, and the top five most frequent combinations were *V3-23/J4*, *V1-2/J4*, *V4-39/J4*, *V3-48/J4* and *V3/7+J4* (Figure 2E). We next explored the differences in length distribution and SHM of heavy-chain CDR3 across major and subpopulations. Although memory B cells exhibited shorter CDR3 lengths than plasma cells (*p* < 0.001) (Figure 2F), they displayed significantly higher SHM levels than both plasma cells (*p* < 0.001) and naive B cells (*p* < 0.001) (Figure 2H). Elevated SHM frequency is typically associated with a more advanced stage of affinity maturation. Further analysis of memory B subsets revealed that the CDR3 length of MB sub2 was significantly longer than that of MB sub1 (*p* < 0.01) and MB sub3 (*p* < 0.001) (Figure 2G), and the SHM levels of MB sub2 (*p* < 0.001) and MB sub3 (*p* < 0.001) were significantly higher than those of MB sub1 (Figure 2I). Recently, identical CDR3 sequences were defined as clones and their repertoire diversity was calculated using Hill numbers [27]. The diversity curves of plasma cells and memory B cells were higher than those of pre-B cells (Figure. 2J). Among the three memory B cell subpopulations, the diversity curves of MB sub2 and MB sub3 were also higher than that of MB sub1. Meanwhile, the Shannon index (*q* = 1) of MB sub2 was significantly higher than that of MB sub1(*p* < 0.001) (Figure 2K), implying that sub2 may harbor rare clones absent in sub1 and sub3. Collectively, these findings suggest that MB sub2 is more likely to contain high-affinity antibodies enriched by affinity maturation.

### 2.3. Localization of Neutralizing Antibodies in Memory B Cell Subsets

**Figure 3:**
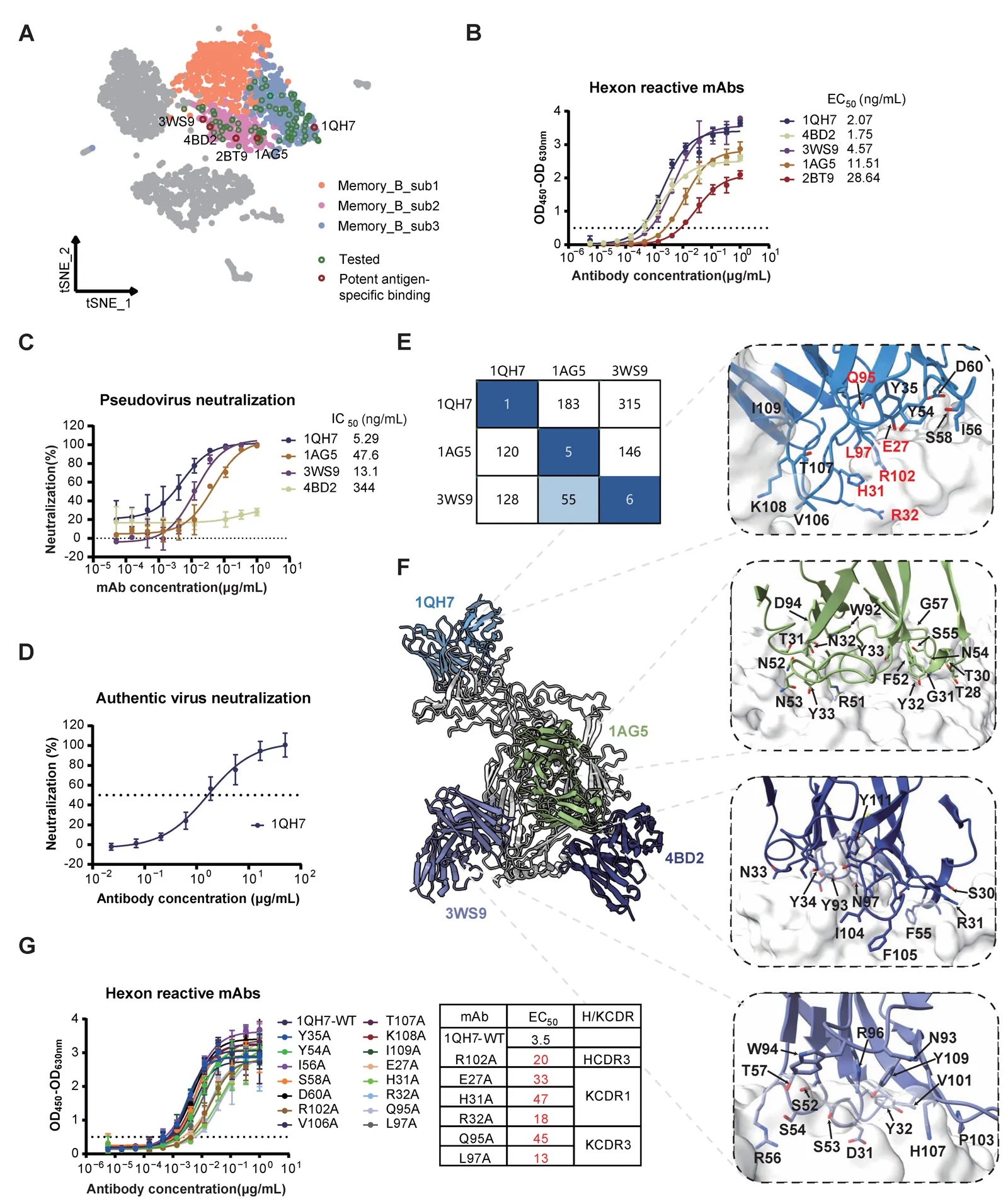
Screening and characterization of antibodies from memory B cell subsets. (**A**) t-SNE visualization of the distribution of memory B cell subsets identified by single-cell sequencing. Red and green dots represent B cells from sub2 and sub3, respectively, to which the B cell receptor sequences belong. (**B**) Detection of antibody binding activity to the HAdV-4 Hexon protein by ELISA. (**C**) Evaluation of antibody neutralizing capacity using a neutralization assay based on the HAdV-4 pseudovirus (Ad4-Luc). (**D**) Validation of neutralization using the authentic HAdV-4 RI67 strain in A549 cells. (**E**) Determination of antibody epitope relationships by competition ELISA. Numbers in the boxes represent the binding percentage of competing antibodies after pre-binding of the primary antibody. An inhibition rate of <33% (dark blue boxes, white numbers) is defined as strongly competitive; >66% (white boxes, black numbers) as non-competitive; and an inhibition rate between 33% and 66% (light blue boxes, black numbers) as an intermediate phenotype. (**F**) AlphaFold3-predicted structures of antibody–Hexon complexes. Structures show antibodies 1QH7 (light blue), 1AG5 (green), 3WS9 (purple), and 4BD2 (dark blue) in complex with the HAdV-4 Hexon protein (gray), highlighting the interaction between the complementarity-determining regions and key amino acid residues. Key amino acid residues within the complementarity-determining regions of each antibody that interact with the Hexon protein are highlighted. (**G**) Alanine-scanning mutagenesis analysis of key binding sites in antibody 1QH7. Binding activity of heavy-chain or light-chain mutants and the wild-type 1QH7 antibody was measured by ELISA, displaying the half-maximal effective concentration (EC_50_) for each antibody–Hexon combination. Antibodies with an EC_50_ > 10 ng/mL are labeled in red. Data in (**B**–**D**,**G**) are presented as the mean ± SD, derived from three technical replicates in a single representative experiment. The results were consistently validated across three independent biological replicates.

Memory B cells represent a prime reservoir for the isolation of high-affinity, potently neutralizing antibodies due to their role in antigen recall and affinity maturation [23]. According to the aforementioned analytical results, several combinations of heavy-chain V and J genes were selected, following the criterion that the CDR length or SHM level in either sub2 or sub3 was higher than that in sub1 (Supplementary Table S2). All BCRs originating from MB sub2 and sub3 with the same VJ usage were synthetically reconstructed for further screening and functional verification. Accordingly, a total of 92 antibody sequences were constructed using the pcDNA3.4 expression vector, and high-affinity antibodies were screened through stepwise functional validation (Figure 3A). First, an enzyme-linked immunosorbent assay (ELISA) identified five monoclonal antibodies, named 1QH7, 4BD2, 3WS9, 1AG5 and 2BT9, with high binding activity to Hexon [28], all exhibiting half-maximal effective concentration (EC_50_) values below 30 ng/mL (Figure 3B). Subsequent pseudovirus neutralization assays, using recombinant adenovirus serotype 4 expressing luciferase (Ad4-Luc), demonstrated that monoclonal antibodies 1QH7, 1AG5, and 3WS9 possessed potent neutralizing activity, with half-maximal inhibitory concentration (IC_50_) values all below 50 ng/mL. Among them, 1QH7 was the most potent (IC_50_ = 5.31 ng/mL) (Figure 3C). Live virus neutralization assays in A549 cells further confirmed that 1QH7 consistently neutralized the authentic HAdV-4 virus (Figure 3D). These results also support the earlier finding that MB sub2 and sub3 are a significant source of such highly efficacious pathogen-specific antibodies.

During the validation process, we noted that the binding antibodies obtained from screening included the IGHV4-61+IGHJ4 and IGHV1-69+IGHJ6 V-J combinations, which is consistent with the HAdV-4-related combinations previously documented in the literature [28]. To explore whether these V-J pairs could yield antigen-binding antibodies outside MB sub2 and sub3, we constructed 44 antibodies from alternative B cell subsets (Supplemental Figure S3A) that utilize these same V-J gene combinations using the pcDNA3.4 expression vector. None of these 44 antibodies showed detectable antigen-binding activity (Supplemental Figure S3B). We note, however, that these control antibodies may differ beyond B cell subset origin, so this observation alone cannot definitively establish subset-intrinsic functional differences. Collectively, our data suggest that MB sub2 and sub3 are a promising reservoir from which potent anti-HAdV-4 antibodies can be isolated.

Given that no therapeutic antibodies against HAdV-4 have been approved for clinical use, this study obtained three human-derived monoclonal antibodies with high neutralizing activity and subsequently conducted an in-depth investigation into their therapeutic potential and mechanism of action. First, to analyze the epitope overlap among the three antibodies, we performed ELISA pairwise competition assays. The results showed that only 1AG5 and 3WS9 exhibited moderate competition (Figure 3E), suggesting that the three antibodies likely bind to distinct epitopes. To further elucidate their recognition mechanisms at the structural level, AlphaFold3 was employed to predict the complex structures of these three antibodies, along with the non-neutralizing control antibody 4BD2, bound to the Hexon protein. Although the fitting performance is suboptimal (Supplementary Table S3), the best-fit model under such conditions can still provide some clues. The predicted models implied that the three antibodies bind to different regions of Hexon, and their epitopes do not overlap with that of 4BD2 (Figure 3F, Supplementary Table S4), which is consistent with the competition assay results. Analysis of the binding interface and interaction patterns based on the structural prediction model suggested that, among the four antibodies, 1QH7 possesses the largest binding interface (Supplementary Tables S3 and S4) and the most extensive interaction network (Supplementary Figure S3), consistent with its stronger binding affinity. This model provides information for subsequent studies on the molecular architecture of antibody–antigen binding. As 1QH7 demonstrated the best neutralizing activity, it was selected for systematic alanine scanning mutagenesis. Totals of 10 and 5 point mutations were introduced into the heavy-chain and light-chain complementarity-determining regions (CDRs), respectively. ELISA binding assays evaluating these mutants revealed that the R102A mutation in the heavy-chain CDR, as well as the E27A, H31A, R32A, Q95A, and L97A mutations in the light-chain CDRs, reduced the antibody’s binding capacity to the antigen (Figure 3G), suggesting these residues might represent key sites for 1QH7-mediated recognition of Hexon.

To further characterize the antibody–antigen interaction, surface plasmon resonance (SPR) was employed to determine the binding kinetics between the Hexon protein and 1QH7. The results demonstrated high-affinity binding with an equilibrium dissociation constant (KD) of 5.88 nM (Supplemental Figure S5A). The thermal stability of 1QH7 was subsequently assessed by differential scanning calorimetry (DSC), which revealed melting temperatures (Tm) of 71.23 °C for the Fab region and 83.45 °C for the CH3 domain, consistent with the typical structural characteristics of an IgG1 [29,30] (Supplementary Figure S5B), indicating favorable structural stability. Furthermore, to evaluate the broad-spectrum neutralizing potential of 1QH7, we tested it against recombinant HAdV-5 and HAdV-7 viruses expressing luciferase. The results showed that 1QH7 exhibited no cross-neutralizing activity (Supplemental Figure S5C,D), confirming its strict serotype specificity. In summary, the monoclonal antibody 1QH7 obtained in this study exhibits high affinity, potent neutralizing activity, strict serotype specificity, and favorable structural stability against HAdV-4.

### 2.4. 1QH7 Protect Against HAdV-4 Challenge in the Stat1-Deficient Mouse Model

**Figure 4:**
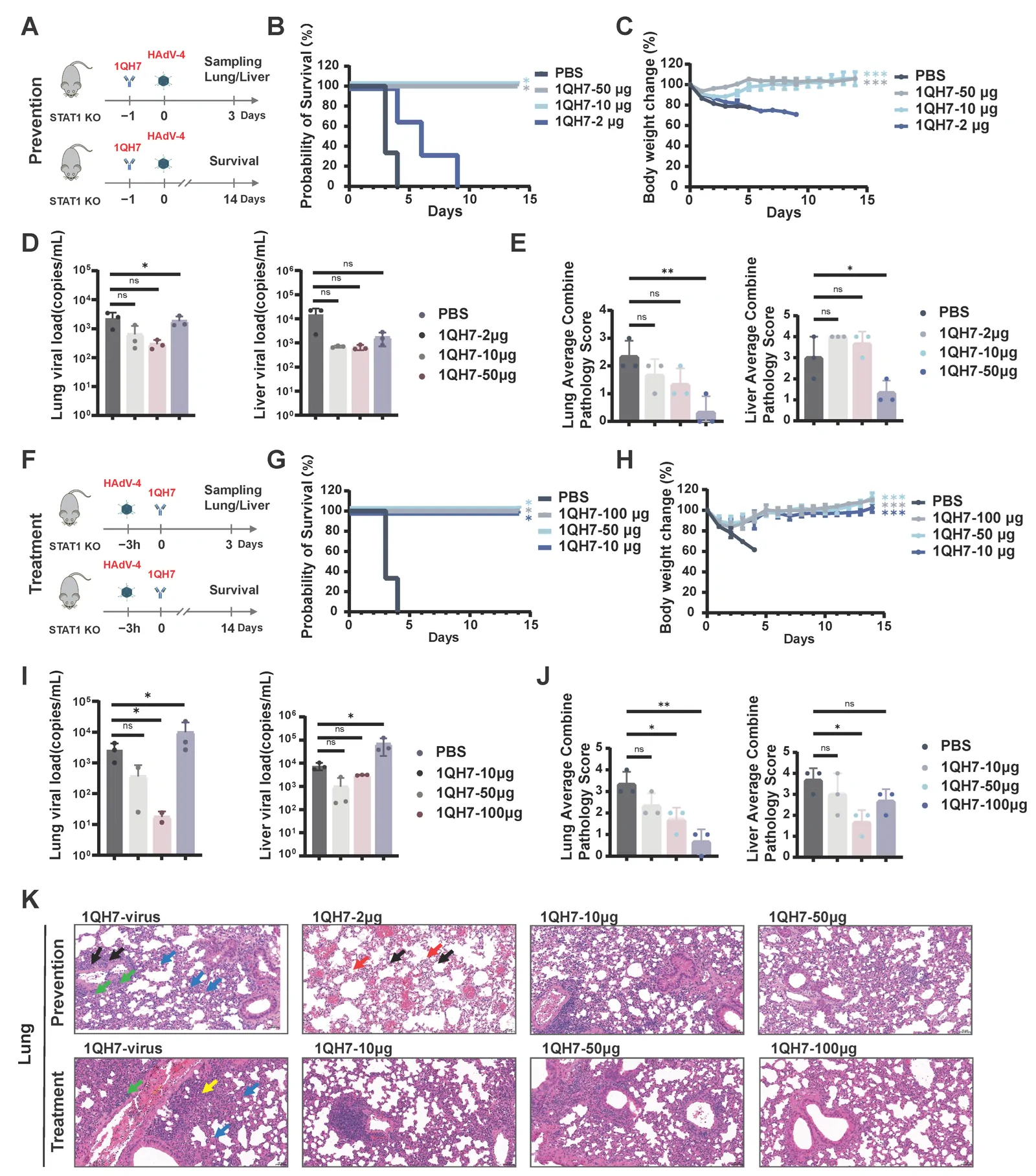
In vivo protective efficacy of antibody 1QH7 in an HAdV-4 infection model using *Stat1*-deficient mice. (**A**) Schematic of the prophylactic immunization regimen. (**B**) Survival curves of mice in the prophylactic group. (**C**) Body weight changes in mice in the prophylactic group. (**D**) Viral loads in lung and liver tissues from the prophylactic group. (**E**) Histopathological scores of lung and liver tissues from the prophylactic group. (**F**) Schematic of the therapeutic immunization regimen. (**G**) Survival curves of mice in the therapeutic group. (**H**) Body weight changes in mice in the therapeutic group. (**I**) Viral loads in lung and liver tissues from the therapeutic group. (**J**) Histopathological scores of lung and liver tissues from the therapeutic group. (**K**) Representative lung tissue sections (H&E staining) from the prophylactic and therapeutic groups. ns (not significant), * *p* < 0.05, ** *p* < 0.01, *** *p* < 0.001.

Based on a previously reported method [29], we evaluated the in vivo protective efficacy of 1QH7 using *Stat1*-deficient mice. *Stat1*-deficient mice were inoculated intraperitoneally with 3 × 10^10^ PFU of authentic HAdV-4. All mice in the virus control group succumbed to infection within 4 days post-challenge, accompanied by significant weight loss. In contrast, prophylactic administration of 1QH7 (2 µg, 10 µg, or 50 µg) 24 h prior to challenge resulted in dose-dependent improvements in survival rates and body weight recovery following transient weight decline (Figure 4A–C). Similarly, post-exposure therapeutic administration of 1QH7 (10 µg, 50 µg, or 100 µg) at 3 h post-infection also yielded dose-dependent enhancements in survival and weight recovery (Figure 4F–H). The 10 µg dose conferred complete protection in both prophylactic and post-exposure therapeutic settings. Viral load analysis of tissues collected at 3 days post-infection revealed that viral copy numbers in the lungs and livers of both the prophylactic and therapeutic groups were significantly lower than those of the virus control group (Figure 4D,I). Pathological examination revealed markedly attenuated lung injury (Figure 4K), and the mean composite pathological scores of the lungs in both the prophylactic and treatment groups were significantly lower than those of the virus control group. (Figure 4E,J). No marked pathological changes were observed in the heart, spleen, or kidneys of either antibody-treated or virus control animals (Supplemental Figure S6A,B), suggesting that these organs are not primary targets of HAdV-4 infection. In summary, 1QH7 exhibited evident protective effects in vivo, supporting its potential utility in both prophylaxis and therapy against HAdV-4 infection.

### 2.5. Mechanism of TRIM21-Mediated Intracellular 1QH7 Neutralization

**Figure 5:**
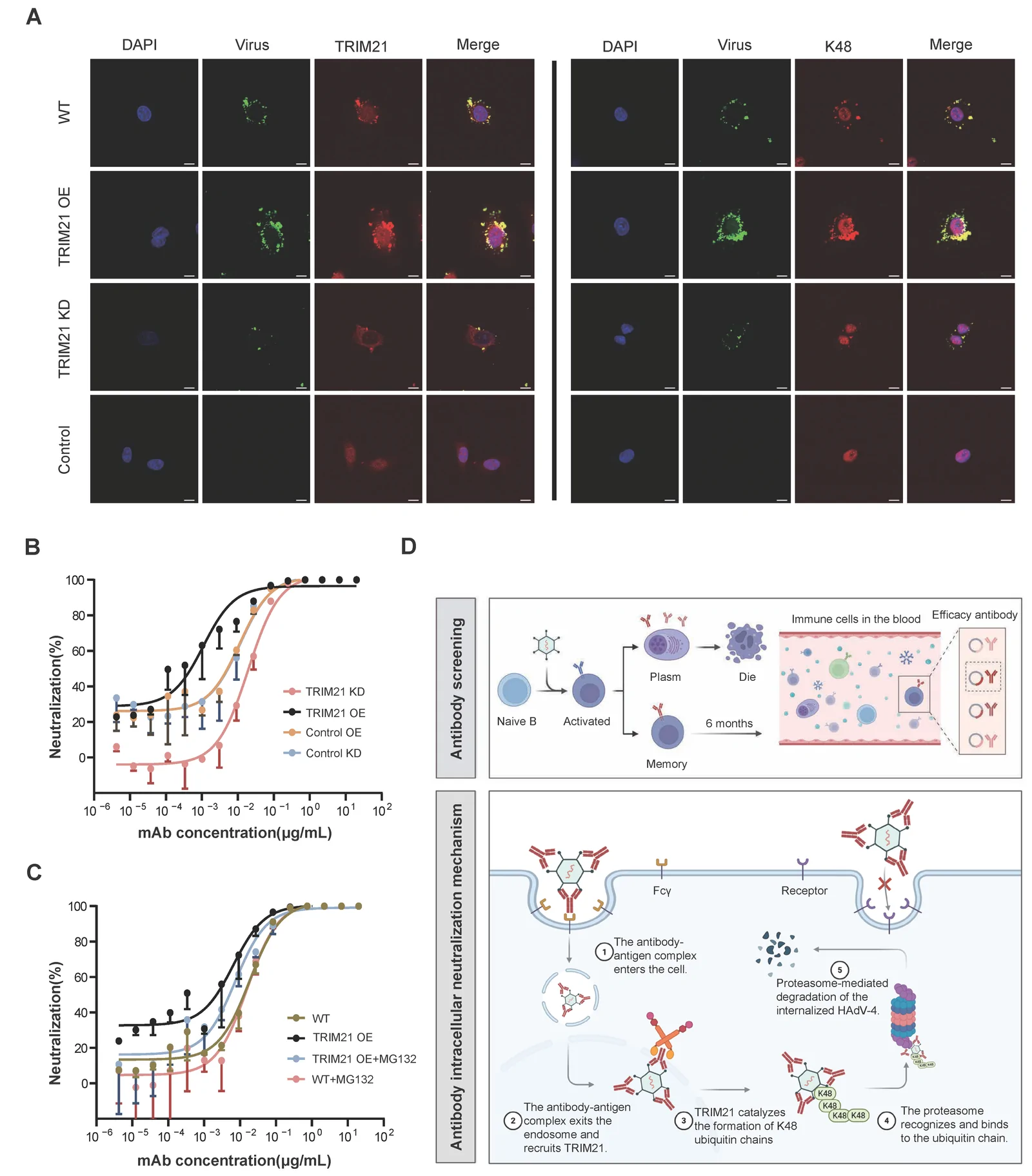
Antibody 1QH7 mediates intracellular neutralization via the TRIM21-dependent ubiquitin–proteasome pathway. (**A**) Confocal microscopy shows intracellular co-localization of 1QH7-precoated HAdV-4 (green, detected with Alexa Fluor 488) and TRIM21 (red, detected with Alexa Fluor 594) in wild-type, TRIM21-overexpressing, and TRIM21-knockdown A549 cells, using a non-neutralizing mAb (4BD2) as a control (left). Intracellular co-localization of 1QH7-precoated HAdV-4 (green) and K48 ubiquitin chains (red, detected with Alexa Fluor 594) (right). Nuclei were counterstained with DAPI (blue). (**B**) Neutralization activity of 1QH7 against HAdV-4-Luc virus in A549 cells with modulated TRIM21 expression (overexpression/knockdown and controls). (**C**) Effect of proteasome inhibition by 1 µM MG132 on 1QH7-mediated neutralization of HAdV-4-Luc in wild-type versus TRIM21-overexpressing A549 cells. All data were shown as mean ± SD of a representative experiment. (**D**) Schematic overview of the 1QH7 antibody screening workflow and the TRIM21-mediated antibody-dependent intracellular neutralization (ADIN) mechanism. Data in (**A**–**C**) are presented as the mean ± SD, derived from three technical replicates in a single representative experiment. The results were consistently validated across three independent biological replicates.

Tripartite motif-containing protein 21 (TRIM21) is a cytosolic E3 ubiquitin ligase that acts as a high-affinity intracellular receptor for the IgG Fc region and plays a crucial role in innate immune responses [31]. To investigate whether the 1QH7 exerts its intracellular neutralizing effect via a similar mechanism, we conducted a series of experiments. Confocal microscopy observation revealed significant co-localization between internalized antibody–virus complexes and endogenous TRIM21 (Figure 5A). Further pseudovirus neutralization assays were performed in cells with TRIM21 knockdown or overexpression. TRIM21 knockdown attenuated antibody-mediated neutralization, whereas TRIM21 overexpression enhanced this activity. These observations support the idea of an important functional role of TRIM21 in this process (Figure 5B). Additional co-localization analysis showed specific recruitment of K48-linked ubiquitin chains to the antibody–virus complexes (Figure 5A). Moreover, the use of ubiquitination pathway inhibitors significantly reduced the antibody’s neutralizing activity (Figure 5C). Taken together, these findings suggest that upon cellular entry, 1QH7 is recognized by the cytosolic receptor TRIM21, which may involve recruitment of the K48-linked ubiquitination system to facilitate intracellular clearance of viral particles in a potential ubiquitin–proteasome-dependent manner (Figure 5D).

## 3. Discussion

During the early stage of an emerging infectious disease outbreak, specific pathogens or pathogen-derived bait proteins are often unavailable for targeted antibody screening. Taking HAdV-4 as an example, this study explored a bait-free antibody-screening strategy based on single-cell RNA and BCR sequencing, leveraging cellular subpopulations to overcome this limitation. HAdV-4 is a significant respiratory pathogen that can cause severe acute respiratory disease (ARD) and fatal outcomes in diverse populations [32,33,34]. In China, neither vaccines nor monoclonal antibody drugs have been approved for marketing against HAdV-4, leaving substantial gaps in prophylactic and therapeutic options.

The advent of single-cell sequencing technology enables precise identification of antigen-specific B cells within infected hosts. By integrating scRNA-seq and scBCR-seq, we can effectively delineate functionally specialized B cell subsets, reconstruct the pairing relationships of light and heavy chains in their native state, and elucidate the in vivo process of affinity maturation. Our analysis revealed three subsets of memory B cells and preliminarily explored their functional differences. Furthermore, the integration of transcriptomic and BCR data highlighted significant enrichment of genes associated with BCR activation and immunoglobulin-mediated immune responses within a specific B cell population, thereby directing our research toward cell types with potential neutralizing activity. In addition, high SHM within the variable region of neutralizing antibodies among various cell population-based expression profiles confirms the antigen-driven evolution of antibodies towards high affinity and specificity. Additionally, the potent highly neutralizing antibody 1QH7 was screened based on the aforementioned results.

This study demonstrates the functional specialization of memory B cell subsets during HAdV-4 infection, aligning with established theories of B cell differentiation [35]. Previous research has documented the clonal expansion of antigen-specific B cells in SARS-CoV-2 and influenza infections [20,23]. Here, we extend this immune paradigm to the HAdV-4 system and validate the strategic value of antibody screening based on functional subgroup characteristics. By integrating scRNA and scVDJ seq, we observed significant transcriptional specialization of B cells post-infection. These findings provide a molecular basis for vaccine design and enhance our understanding of the in vivo immune response mechanisms.

It is well known that monoclonal antibodies to HAdV are generally serotype-specific, and previous studies have shown little or no cross-reactivity with other HAdV species [36]. This study also demonstrated that 1QH7 exhibited high specificity for HAdV-4 and did not display reactivity in cross-neutralization assays against HAdV-5 and HAdV-7 from different species, confirming that it is a serotype-specific antibody. Moreover, the antibody demonstrated high in vitro affinity (KD = 5.88 nM), excellent thermal stability, and potent inhibitory activity against HAdV-4 infection at low concentrations (IC_50_ = 5.29 ng/mL). These properties are inferior to those of the reported antibody 2CF4 (IC_50_ = 0.34 ng/mL; KD not reported) [28] and NVA17 (IC_50_ = 2.67 ng/mL; KD = 1.742 nM) [31]. Notably, similar VJ gene combinations exhibited a consistent moderate neutralization ability based on sequence homology, suggesting an intrinsic correlation between gene usage preferences and functional outcomes for the same virus infection [28]. In this study, the protective effect of 1QH7 in vivo was verified in a *Stat1*-deficient mouse model, where our proprietary antibody 1QH7 conferred complete prophylactic and therapeutic protection against viral infection in mice at a concentration of 10 μg/mL. In comparison, antibody 2CF4 was capable of providing prophylactic protection at a lower concentration of 2 μg/mL [28]. In contrast, NVA17 required a substantially higher concentration of 50 μg/mL to achieve full prophylactic and therapeutic protection against the viral challenge in mice [31]. Although the virus-challenge model enables rapid evaluation of antibody efficacy, it does not fully recapitulate natural respiratory infection. In addition, due to the limitations of experimental design, only PBS control was set in this study without an isotype-matched irrelevant IgG control. Therefore, Fc-mediated effector functions cannot be completely excluded as contributors to the observed protective effects, and follow-up studies with supplementary IgG control groups are needed to further dissect the underlying protective mechanisms of 1QH7.

TRIM21 is known to act as a high-affinity Fc receptor that facilitates antibody-mediated neutralization via proteasomal degradation. Consistent with this established pathway, our data suggest that TRIM21 expression modulates the colocalization of internalized 1QH7 with viral particles. The colocalization of K48-ubiquitin chains and the antibody–virus complex suggests that proteasomal degradation is involved in antibody neutralization. The partial reduction in neutralizing activity upon MG132 treatment further supports a potential contribution of TRIM21 to this pathway. These results suggest that TRIM21 may act as a mediator of antibody-dependent intracellular neutralization, providing a plausible mechanistic basis for the development of targeted antiviral strategies.

This study establishes an integrated single-cell sequencing framework to dissect the B cell immune response to HAdV-4 infection. By combining scRNA-seq and scVDJ-seq, we successfully characterized the transcriptional and BCR repertoire landscape of B cells at unprecedented resolution. Our analysis not only revealed shifts in B cell composition following infection and recovery, but also identified functionally specialized memory B cell subsets, with MB sub2 and MB sub3 subsets exhibiting transcriptional signatures associated with potential neutralizing antibody production. Based on these findings, we synthesized 92 antibodies corresponding to the specific V-J gene combinations enriched in this subpopulation for expression and screening, and successfully identified three neutralizing antibodies. Among them, the monoclonal antibody 1QH7 demonstrated potent neutralizing activity in both cellular and animal models. Further integration of antigen–antibody structural analysis and confocal microscopy-based co-localization studies of intracellular neutralization provided initial insights into its mechanism of action. Moreover, the other two neutralizing antibodies could be utilized to develop combination therapies or bispecific antibodies, offering additional options for future therapeutic strategies.

While this study demonstrates the efficacy of integrated single-cell technology in antibody discovery, several limitations remain that require further improvement. The B cell sorting strategy utilizing flow cytometry may favor subsets with high surface BCR expression. Future work could incorporate single-cell antigen labeling techniques, such as LIBRA-seq, to directly link BCR sequences with antigen specificity without pre-sorting. Additionally, our focus on a single donor limits the generalizability of findings regarding germline gene usage preferences. Expanding the donor sample size will facilitate the identification of conserved BCR features associated with neutralization breadth and aid in the development of broad-spectrum antibodies.

## 4. Materials and Methods

### 4.1. Cells and Culture Conditions

Human lung carcinoma A549 (No. CCL-185) cells and human embryonic kidney 293 (HEK293, No. CRL-1573) were purchased from American Type Culture Collection (ATCC) and cultured in Dulbecco’s Modified Eagle Medium (DMEM; Gibco, Waltham, MA, USA) supplemented with 10% fetal bovine serum (FBS; Excell Bio, Shanghai, China), 2 mM GlutaMAX™ supplement (Gibco), and 1% penicillin-streptomycin (100 IU/mL) at 37 °C in a humidified 5% CO_2_ atmosphere. Expi293F cells were obtained from Thermo Fisher Scientific, Waltham, MA, USA (R79007) and cultured in FreeStyle™ 293 Expression Medium (Gibco) at 37 °C under 5% CO_2_ in a Multitron-Pro shaker (Infors HT, Bottmingen, Switzerland). All cell lines used in this study were routinely tested for mycoplasma contamination.

### 4.2. Viruses

The HAdV4-RI67 strain (GenBank: AY594253.1) and recombinant viruses (HAdV4/5/7-Luc) used in the experiments were obtained from our laboratory. HAdV4-RI67 was propagated in Expi293F cells using FreeStyle™ 293 Expression Medium (Gibco) at 110 rpm and 37 °C in a humidified atmosphere with 5% CO_2_. When 70% to 90% of the cells exhibited typical cytopathological effects (CPEs) of viral infection (approximately 72 h), the cells underwent three freeze–thaw cycles to achieve lysis. Cell lysates were centrifuged at 5000× *g* for 20 minutes at 4 °C, pellets of cellular debris were discarded, and supernatants were concentrated using Amicon^®^ Ultra-15 Centrifugal Filters with an Ultracel^®^-100 kDa membrane (Merck millipore, Burlington, MA, USA). Infectious virions were titrated and recorded as median tissue culture infectious dose (TCID_50_) as described in [37]. To facilitate dose comparison with previous adenovirus challenge studies, challenge doses are reported in PFU, derived from the standard Poisson-based conversion PFU/mL = 0.7 × TCID_50_/mL.

The E3 region of the recombinant viruses HAdV4-Luc, HAdV5-Luc, and HAdV7-Luc was replaced with the luciferase gene. HEK293 cells were infected at a multiplicity of infection (MOI) of 5 and cultured at 37 °C under 5% CO_2_ for 48 h. When approximately 90% of the cells exhibited typical cytopathic effects (CPEs), both the cells and culture supernatant were collected. The samples were lysed through three freeze–thaw cycles, followed by centrifugation at 5000× *g* for 20 min at 4 °C to remove cellular debris. The supernatant was concentrated using an Amicon^®^ Ultra-15 centrifugal filter unit equipped with a 100 kDa ultrafiltration membrane, after which the viral titer was determined. All viruses were stored at −80 °C.

### 4.3. B Cell Sorting

PBMCs were isolated from Chinese convalescent donors one month after natural HAdV-4 infection between 3 and 14 January 2022, as previously described [28]. Separation of PBMCs from collected blood samples was accomplished through density gradient centrifugation using Ficoll according to the manufacturer’s instructions (DKW-LST-25050SK). Briefly, blood samples were slowly transferred above equal-volume lymphocyte separation medium. After centrifugation at 800× *g* for 30 min, PBMCs were collected, washed twice with PBS, resuspended in cell freezing medium (90% FBS and 10% DMSO), and preserved at −80 °C [28].

PBMCs were stained with fluorescently labeled antibodies, including anti-CD19-Alexa Fluor 700 (302226, BioLegend, San Diego, CA, USA) and anti-CD3-PerCP (300326, BioLegend, San Diego, CA, USA), at 4 °C for 1 h. After staining, the cells were washed twice with cell staining buffer. B cells were then sorted using an MA900 cell sorter (SONY, San Jose, CA, USA), with target cells defined as CD3^−^ CD19^+^. The research with human samples was approved by the Ethics Committee of GIBH, and informed consent was obtained from each volunteer.

### 4.4. Single-Cell RNA-Seq and VDJ-Seq Library Preparation and Sequencing

Single-cell RNA-seq and VDJ-seq libraries were constructed from the sorted PBMC following the manufacturer’s standard protocol based on the Chromium Next GEM Single Cell 5′ Reagent Kit v2 (Dual Index) (10x Genomics, PN-CG000331 Rev F). The core kit components included the Chromium Next GEM Single Cell 5′ Kit v2 (PN-1000263), Library Construction Kit (PN-1000190), Chromium Single Cell Human BCR Amplification Kit (PN-1000253), Chromium Next GEM Chip K Single Cell Kit (PN-1000286), and Dual Index Kit TT Set A (PN-1000215) for dual-indexed library labeling.

Briefly, the qualified single-cell suspension was loaded onto the Chromium Next GEM Chip K, and oil-in-water gel beads-in-emulsion (GEMs) were generated on the Chromium Controller to achieve single-cell barcoding, with the GEM reverse transcription (GEM-RT) process proceeding without abnormalities. After GEM-RT incubation, the post GEM-RT product was purified and full-length cDNA was amplified via PCR with cycle numbers optimized. The amplified cDNA was quantified and gene expression library construction was performed via enzymatic fragmentation, end repair, A-tailing, and adaptor ligation. For BCR V(D)J library preparation, target enrichment was conducted from the amplified cDNA using the human BCR-specific primer mixes (PN-2000254, PN-2000255) with two rounds of Nested PCR amplification and library construction steps consistent with the 10x Genomics protocol. High-throughput sequencing was performed on the libraries on an Illumina sequencing platform with paired-end dual-index sequencing.

### 4.5. Sequencing Data Processing

The workflow was processed based on Cell Ranger (v8.0.1) for the assembly and annotation of the scRNA seq raw sequencing data. The GRCh38 human reference genome from the 10× Genomics official website was the reference; Then, generated matrices were loaded and merged into a single Seurat object. Downstream dimensionality reduction, clustering, and cell annotation were based on the Seurat (v 5.1.0) package with R (v 4.4.1) following data cleaning. After LogNormalize normalization, 2000 highly variable features (HVFs) were selected via the “vst” method. Cell cycle effects (S/G2M scores) were regressed out during scaling of HVFs. PCA was performed on scaled HVFs, and biologically relevant PCs were identified by Jackstraw permutation test and Elbow plot. Cells were clustered using KNN graph-based methods (resolution = 0.5) and visualized via t-SNE. The scVDJ-seq raw fastq files were processed using the 10X Genomics CellRanger (v8.0.1) pipeline. Contig assembly, annotation, and clonotype analysis were performed using ‘‘cellranger vdj’’ with the Cell Ranger V(D)J compatible reference.

The cell annotation among different clusters was based on the marker gene derived from a previous study. Differentially expressed genes (DEGs) were identified using the FindMarkers( ) function, with the filtering parameters set as min.pct = 0.25 and logfc.threshold = 0.25. GO enrichment analysis for DEGs was performed with enrichGO( ) from the clusterProfiler package (v 4.12.0), using the “org.Hs.eg.db” annotation database (human) for annotation. Pseudotime trajectory analysis was performed with the Monocle package (v2.32.0). SHM was performed by mapping the VDJ sequence against the germline reference originated from the international ImMunoGeneTics information system (IMGT). The diversity of BCR was calculated using Alakazam (v 1.4.2). R Package scop (v 0.8.1), ggpubr (v 0.6.0), pheatmap (v 1.0.12) and ggplot2 (v 3.5.1) were used for data visualization.

### 4.6. Hexon Protein Expression and Purification

For use in flow cytometry, ELISA and SPR, the Hexon protein was produced by infecting Expi293F cells with the HAdV4-RI67 strain. The detailed procedure was as follows: Expi293F cells were infected with HAdV-4 and cultured under saturated humidity at 37 °C, 110 rpm, and 5% CO_2_. Approximately 72 h post-infection, when 70% to 90% of the cells exhibited cytopathic effects (CPEs), the cells were harvested by centrifugation at 5000× *g* for 20 min at 4 °C. After discarding the cell debris, the supernatant was subjected to initial purification using a Core 700 size-exclusion column equilibrated with a buffer containing 20 mM Tris and 150 mM NaCl (pH 7.5). The elution peak corresponding to the target protein was collected based on UV absorbance monitoring. This fraction was concentrated using a 100 kDa ultrafiltration device, followed by further purification on a Superdex 200 size-exclusion column using PBS as the mobile phase. The target protein peak was again collected according to UV monitoring. The final protein concentration was determined using a NanoVue Plus spectrophotometer, and the purified protein was aliquoted and stored at −80 °C.

### 4.7. Antibody Preparation

Variable region nucleotide sequences of paired heavy- and light-chain BCRs from each immunization group were selected, codon-optimized and full-length antibody sequences were cloned into pcDNA3.4 using XbaI and HindIII restriction sites, and signal peptide and Kozak sequences were added to the N-terminal of expressed sequences. Plasmids of paired heavy- and light-chain genes were co-transfected into the Expi293F cells (Thermo Fisher Scientific) according to the manufacturer’s protocol. The supernatants were harvested by centrifugation and subsequently filtered through 0.22 µm syringe filters (PALL, Bristol, UK, Cat# 4612) or disposable vacuum systems (Biosharp, Hefei, China, Cat# BS-500-XT). Recombinant proteins were purified on an ÄKTA Pure 150 purification system (GE Healthcare, Piscataway, NJ, USA) using a 5 mL Protein A column (Cytiva, Marlborough, MA, USA; binding buffer: PBS, pH 7.5; elution buffer: 0.1 M Glycine, pH 2.7) [16]. All proteins were analyzed for purity by SDS–PAGE, and the concentration was determined using NanoVue Plus (GE Healthcare) or the UV 280 nm absorption method.

### 4.8. Assessment of Antibody Binding Activity by ELISA

Purified Hexon-antigen proteins (2 μg/mL) were immobilized on ELISA 96-well plates (Corning, New York, NY, USA) overnight in a sodium bicarbonate buffer (50 mM sodium carbonate, 50 mM sodium bicarbonate, pH 9.6) at 4 °C. Plates were washed with PBST (PBS + 0.2% Tween 20) and blocked with blocking buffer (PBS + 2% BSA) for 1 h at 37 °C. Blocking buffer was removed and plates were washed with PBST (PBS + 0.02% Tween 20). After washing with PBST (PBS + 0.02% Tween 20), monoclonal antibodies were assessed starting at a concentration of 1 μg/mL and subjected to 11 additional threefold serial dilutions; 100 µL of the antibodies was added to each well (100 µL/well) and incubated for 1 h at 37 °C. The plates were washed and then incubated (100 µL/well) with an HRP-conjugated anti-human IgG Fc secondary antibody (Abcam97225, Cambridge, UK) at a dilution of 1:10,000. The plates were washed again with PBST, TMB single-component substrate solution (Solarbio, Beijing, China, Cat# PR1200) was added to each well of the plate (100 µL/well) and incubated in the dark at room temperature for 5 min, and the reaction was stopped using 2 M H_2_SO_4_ (50 µL/well). The absorbance was read at 450 nm/630 nm using a microplate reader (Spectra Max 190, Molecular Devices, San Jose, CA, USA).

### 4.9. Competition-Binding ELISA

First, 200 μg of the antibody was incubated overnight at 4 °C with a 5-fold excess of biotin. Biotinylated antibodies were PBS-exchanged several times to remove excess biotin, and the concentration was determined using NanoVue Plus (GE Healthcare) at 280 nm. The competition-binding ELISA was performed in 96-well plates. Purified Hexon proteins (2 µg/mL) were immobilized on ELISA plates overnight in a sodium bicarbonate buffer (50 mM sodium carbonate, 50 mM sodium bicarbonate, pH 9.6) at 4 °C. Plates were washed with PBST (PBS + 0.02% Tween 20) and blocked with blocking buffer (PBS + 2% BSA) for 1 h at 37 °C. Blocking buffer was removed and plates were washed with PBST (PBS + 0.02% Tween 20). The first antibody (20 µg/mL) in antibody diluent buffer (PBS + 0.2% BSA+ 0.01% Tween 20) was added at 50 µL/well and incubated for 1 h at 37 °C. The biotinylated second antibody (1 µg/mL; final concentration of 0.5 µg/mL) in antibody diluent buffer (PBS + 0.2% BSA + 0.01% Tween 20) was added at 50 µL/well and incubated for 1 h at 37 °C. Plates were washed with PBST (PBS + 0.02% Tween 20) and incubated with an HRP-conjugated streptavidin (Abcam, Cat# AB7403) for 1 h at 37 °C. Plates were washed again. The TMB single-component substrate solution (Solarbio, Cat# PR1200) was added to each well of the plate (100 µL/well) and incubated in the dark at room temperature for 5 min. The reaction was stopped with the addition of 2 M H_2_SO_4_ (50 µL/well), and the absorbance was read at 450 nm/630 nm using a microplate reader (Spectra Max 190, Molecular Devices). The percentage of bound biotinylated antibodies was calculated by comparing the absorbance value in the presence of competitors to that in the presence of an irrelevant control Nb(S309). Antibodies were considered to be competing for the same or close epitopes if the percentage of bound detected Nb was <33%. Antibodies were assumed to bind to different sites if the percentage value was >66%. A group of intermediate competitive antibodies was identified if their percentage value was in the range of 33–66% [38].

### 4.10. Pseudotyped Virus Neutralization Assay

Recombinant viruses (HAdV4/5/7-Luc) were constructed by replacing the E3 regions with a luciferase expression cassette. To perform the neutralizing assay, 50 μL of recombinant virus was incubated with 50 μL of threefold serially diluted antibody (starting concentration at 1 μg/mL) in 96-well cell culture plates at 37 °C for 1 h followed by the addition of 2.5 × 10^4^ A549 cells in 100 μL of DMEM supplemented with 10% FBS into each well. The plates were incubated at 37 °C and 5% CO_2_ for 24 h. After incubation, 200 μL of the medium was removed, and 100 μL of Luciferase Assay cell culture Lysis 5× Reagent (Promega) was added to each well and incubated for 10 min at 37 °C. After incubation, each well was mixed 10 times by pipetting, and 20 μL of the mixture was transferred to a white plate to measure the relative light unit (RLU) using a microplate reader (Promega).

To investigate the role of TRIM21 in antibody-mediated intracellular neutralization, the same assay was performed using TRIM21-knockdown, TRIM21-overexpressing A549 cells, and their corresponding empty vector controls instead of wild-type cells. The neutralizing efficiencies across these genetically modified cell lines were compared.

To examine the dependency of the neutralization process on the proteasomal pathway, the proteasome inhibitor MG132 (1 µM) was added to the culture wells at the time of seeding with either wild-type or TRIM21-overexpressing A549 cells. Following a 24 h incubation at 37 °C, luciferase activity was measured and compared with that of untreated control groups.

### 4.11. Authentic Virus-Neutralizing Assay

All processes in this study involving authentic HAdV-4 were performed in a biosafety level 2 (BSL-2) facility. The neutralizing activity of antibodies against authentic HAdV4-RI67 was assessed using a microneutralization assay as previously described [39,40]. Briefly, 3 × 10^4^ A549 cells in 100 μL DMEM medium with 10% FBS were inoculated into each well of 96-well cell culture plates and grown overnight at 37 °C and 5% CO_2_. Antibodies (initial concentration at 50 μg/mL) were serially diluted threefold and mixed with 100 TCID_50_ of HAdV-4, followed by incubation at 37 °C for 1 h. After discarding the cell culture supernatants, 200 μL of the virus–antibody mixture was added to the A549 cells and incubated with 5% CO_2_ at 37 °C for 3 days. A microscope was used to observe the infected cells and then the viability of the A549 cells was determined using a Cell Counting Kit-8 (CCK-8) assay (Vazyme Biotech, Nanjing, China) according to the manufacturer’s protocol. After washing the 96-well cell culture plate twice with sterile PBS, DMEM medium (containing 2% FBS and 10% CCK-8 reagent) was added at 100 μL/well and incubated for 30 min at 37 °C in a cell incubator. The absorbance value at 450 nm was then measured using a microtiter plate reader. The neutralizing titers and concentrations of the mAbs were used to calculate the IC_50_.

### 4.12. In Vivo Animal Challenge Experiment

Female *Stat1*-deficient mice (6–8 weeks old) were used in this study. Sample sizes were set at n = 3 per group. Mice were randomly assigned to experimental groups using simple randomization. Endpoint assessments and histopathological evaluations were performed in a blinded manner. The predefined humane endpoints included a body weight loss of ≥25%, severe impairment of mobility, or a moribund state, at which point animals were euthanized. The experiment comprised prophylactic and post-exposure therapeutic groups. All mice were anesthetized and challenged via intraperitoneal (i.p.) injection with 3 × 10^10^ PFU of HAdV4-RI-67. Mice in the prophylactic group received i.p. injections of 1QH7 at graded doses (50 µg, 10 µg, or 2 µg) 24 h prior to challenge, whereas mice in the post-exposure therapeutic group received i.p. injections of 1QH7 at graded doses (100 µg, 50 µg, or 10 µg) at 3 h post-infection. Body weight and survival were monitored and recorded daily for 14 consecutive days post-challenge. On day 3 post-infection, lung and liver tissues were collected. A portion of the tissues was fixed in 10% formalin, embedded in paraffin, sectioned, and subjected to hematoxylin and eosin (H&E) staining for histopathological examination [41]. Genomic DNA was extracted from another portion of the tissues using the QIAamp DNA Mini Kit, and viral load was quantified by SYBR Green-based real-time quantitative PCR (qPCR). The primer sequences used were: HAdV-4-F: 5′-CAAGGACTACCAGGCCGTCA-3′ and HAdV-4-R: 5′-GTTAGCATAGAGCATGTTCT-3′. The thermal cycling protocol was as follows: 95 °C for 5 min; 40 cycles of 95 °C for 30 s, 55 °C for 30 s, and 72 °C for 30 s; followed by a melt curve analysis from 65 °C to 95 °C with an increment of 0.5 °C per cycle for 5 s. Viral genome copy numbers were quantified based on a standard curve generated from serial dilutions of genomic DNA extracted from the virus stock. All animal experimental protocols were approved by the Institutional Animal Care and Use Committee (IACUC) of the Academy of Military Medical Sciences and conducted in accordance with standard operating procedures within a Biosafety Level 2 (BSL-2) facility.

### 4.13. Surface Plasmon Resonance (SPR) Assay

Antibody–antigen binding kinetics were determined by SPR technology using a Biacore T200 instrument (Cytiva). Experiments were performed at a flow rate of 30 µL/min and a temperature of 25 °C using HBS-EP (0.01 M HEPES pH 7.4, 0.15 M NaCl, 3 mM EDTA, 0.005% *v*/*v* Surfactant P20) as running buffer. First, the antibody was diluted to 1 µg/mL and captured by the Protein A chip at a flow rate of 10 µL/min. The antibody was diluted in HBS-EP ^+^ buffer (GE Healthcare) to a concentration of 0.5 μg/mL and then subjected to a Protein A chip at a flow rate of 10 μL/min for 60 s. Then, the purified Hexon protein was injected at concentrations of 100 nM, 50 nM, 25 nM, 12 nM, 6.25 nM, and 0 nM, passed through the Protein A chip that had captured the antibody at a flow rate of 30 µL/min. The flow durations were 120 s for the association stage and 900 s for dissociation. The chip surface was regenerated between each cycle using 10 mM glycine at pH 1.5. Sensorgrams for the association and dissociation phases were fitted using Biacore Insight Evaluation software version 5.0.18.22102 (Cytiva).

### 4.14. Differential Scanning Calorimetry (DSC) Assay

Antibody thermodynamic stability was determined by DSC technology using a Nano DSC instrument (TA). We ran DSCRun™ software (version 3.11.0) on the computer and initial thermal stabilization took one hour. The antibody sample was diluted in PBS (Gibco) to a concentration of 1 mg/mL and the reference (PBS) had to be degassed for 10 min using a degassing station (TA). We started the program after pressurizing the equipment to 45 psi (3 atm), the heating rate was 1 °C per minute, the temperature range was from 25 to 100 °C, and the initial equilibrium time was 600 s. Multiple scans were carried out to evaluate the repeatability equilibration period [30].

### 4.15. Simulation of Structure of Antigen–Antibody Complexes Through Alphafold3

The amino acid sequence of the adenovirus Hexon antigen protein (GENBANK: AAT97450.1) was obtained from the GENBANK gene sequence database (https://www.ncbi.nlm.nih.gov/genbank/ (accessed on 3 June 2022)), and the amino acid sequences of heavy chains and light chains of selected monoclonal antibodies were obtained at the same time. The Hexon antigen protein sequences and heavy-chain and light-chain sequences of monoclonal antibodies were input into localized AlphaFold 3.0.0 software for prediction of antigen–antibody complex structure. Default model parameters are used in the process of structure prediction, and various confidence indexes provided by AlphaFold 3.0.0 are used to evaluate the prediction model. The local confidence score (pLDDT), prediction alignment error (PAE), prediction template modeling score (pTM) and interface prediction template modeling score (ipTM) are synthesized. The model with the highest ranking score is selected as the final structure, and the ChimeraX 1.8 software is used for visual analysis. The interaction interface and spatial conformation between antigen and antibody are demonstrated [42]. The superposition of different protein complex structures was illustrated using the “Structure Analysis-Matchmaker” tool in UCSF ChimeraX version 1.8.

### 4.16. Immunofluorescence Assay

Wild-type, TRIM21-overexpressing, and TRIM21-knockdown A549 cells were seeded on coverslips in 24-well plates at a density of 5 × 10^4^ cells/well and cultured overnight at 37 °C for adhesion. Prior to infection, cells were gently washed twice with DMEM. Ad4-RI67 virus (1 × 10^8^ PFU) was mixed with 10 µg of 1QH7 (negative control: non-neutralizing nanobody 4BD2) in 500 µL of DMEM and pre-incubated at room temperature for 30 min. After removing the culture medium, 500 µL of the virus–antibody mixture was added to each well and incubated at 37 °C for 6 h. Post-infection, cells were washed three times with PBS, fixed with 4% paraformaldehyde for 20 min at room temperature, permeabilized with 0.5% Triton X-100 for 15 min, and blocked with PBS containing 5% BSA and 0.1% Tween-20 for 1 h. For immunostaining, cells were incubated with a rabbit anti-TRIM21 primary antibody (1:200 dilution) at 4 °C for 1 h, followed by three washes with PBST. Subsequently, the cells were incubated with Alexa Fluor 594-conjugated goat anti-rabbit secondary antibody (1:400) and DyLight 488-conjugated goat anti-human secondary antibody (1:1000) for 30 min each at room temperature in the dark, with three PBST washes after each secondary antibody incubation. Nuclei were stained with DAPI (1:2000 dilution) for 10–15 min. All fluorescence images were acquired using a Zeiss Axio Observer microscope (Carl Zeiss Microscopy GmbH, Jena, Germany) equipped with a 63× objective.

### 4.17. Statistical Analysis

For the ELISA, the EC_50_ values for monoclonal antibodies were determined using four-parameter nonlinear regression (GraphPad Prism v10.2.3). For the HAdV-4-Luc recombinant virus neutralizing assay, the inhibition percentage was calculated by comparing the relative luminescence units to the blank control (cells without the pseudotyped virus or antibody) and virus control (cells with the pseudotyped virus but without the antibody). IC_50_ was calculated using a three-parameter nonlinear regression (GraphPad Prism v10.2.3). For the authentic-virus neutralizing assay, neutralization percentage was calculated as (Sample signals − Virus control signals)/(Blank control signals − Virus control signals) × 100%. Data were fitted using a three-parameter nonlinear regression (GraphPad Prismv10.2.3). For the SPR assay, affinity values, including association rates (Kon), dissociation rates (Koff), and affinity constants (K_D_), were calculated using Biacore T200 Evaluation Software v3.2.2 with a 1:1 binding model. For the DSC assay, we used Nano Analyze data analysis software v4.0.1 to accurately fit the Nano DSC data into the model and calculate the unfolding temperature, unfolding entropy, and unfolding enthalpy.

Schematic diagrams of animal immunization experiments and illustrations depicting the intracellular neutralization mechanism of antibodies were created using the online platform Biorender. For the antigen–antibody complex structure, conformational prediction was first performed using AlphaFold 3. Based on the predicted model, the binding interface was analyzed with PDBePISA, interacting residues were predicted via the PDBsum server, and structural visualization of the complex was finally generated using Chimera X1.8 software. A two-tailed Student’s *t*-test was used when comparing data between two groups, and a one-way analysis of variance (ANOVA) was used for multiple comparisons between more than two groups. Kaplan–Meier survival curves were constructed, and statistical differences between groups were determined by the log-Rank test. Multiple-testing correction was performed using the Benjamini–Hochberg FDR approach to restrict the false-discovery rate to ≤1%. Body weight changes were analyzed by two-way ANOVA (mixed-effects model) followed by Dunnett’s post hoc test for comparisons against the virus control group. For viral load and histopathological scores, data were evaluated by ordinary one-way ANOVA with Dunnett’s multiple comparisons test. Statistical analyses were performed using GraphPad Prism v10.2.3. Results are expressed as the mean ± SD. Statistical significance was considered at * *p* < 0.05, ** *p* < 0.01, and *** *p* < 0.001.

## Data Availability

The RNA sequencing data generated in this study have been deposited in the National Genomics Data Center (NGDC) Genome Sequence Archive under BioProject accession code [PRJCA057286]. The original contributions presented in the study are included in the article. Further inquiries can be directed to the corresponding author.

## Supplementary Materials

The following supporting information can be downloaded at: https://www.mdpi.com/article/10.3390/ijms27208952/s1.
