# Supplementary material for: Single-Cell BCR and Transcriptome Analysis Revealed Potent Neutralizing Antibodies in Response to HAdV-4 Infection
Source: Int J Mol Sci. 2026 Oct 9;27(20):8952. doi: 10.3390/ijms27208952 (PMC13704714; doi:10.3390/ijms27208952)
Supplement: Supplementary file 1 [file ijms-27-08952-s001.zip › ijms-4575000-supplementary.pdf]

Supplementary Materials

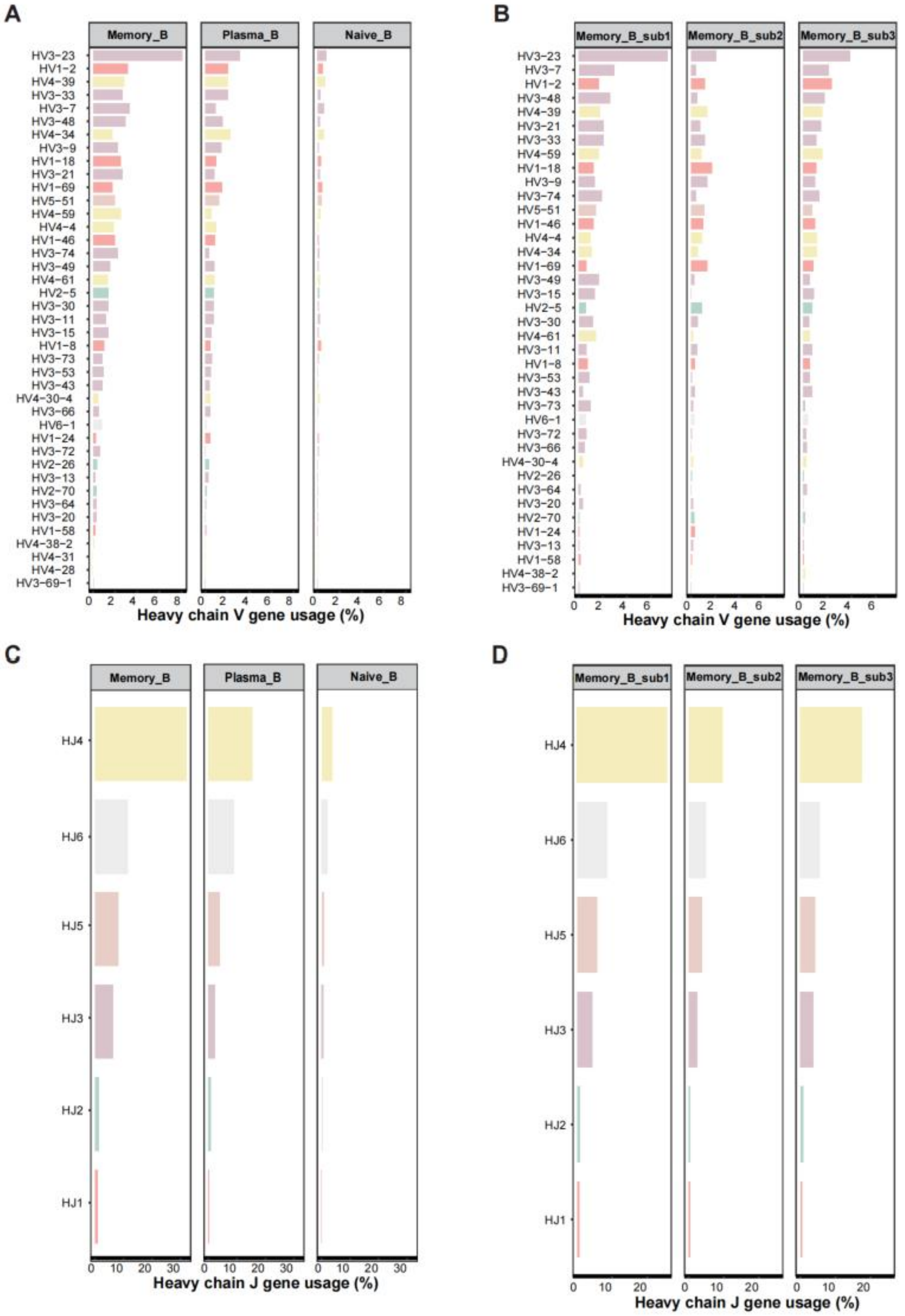

Supplemental Figure S1. The proportion of various V and J gene usage in Heavy chain among various B cells. A. V gene usage of heavy chains among major type of B cells. **B.** V gene usage of heavy chains among memory B cell subpopulations. **C.** J gene usage of heavy chains among major type of B cells. **D.** J gene usage of heavy chains among memory B cell subpopulations.

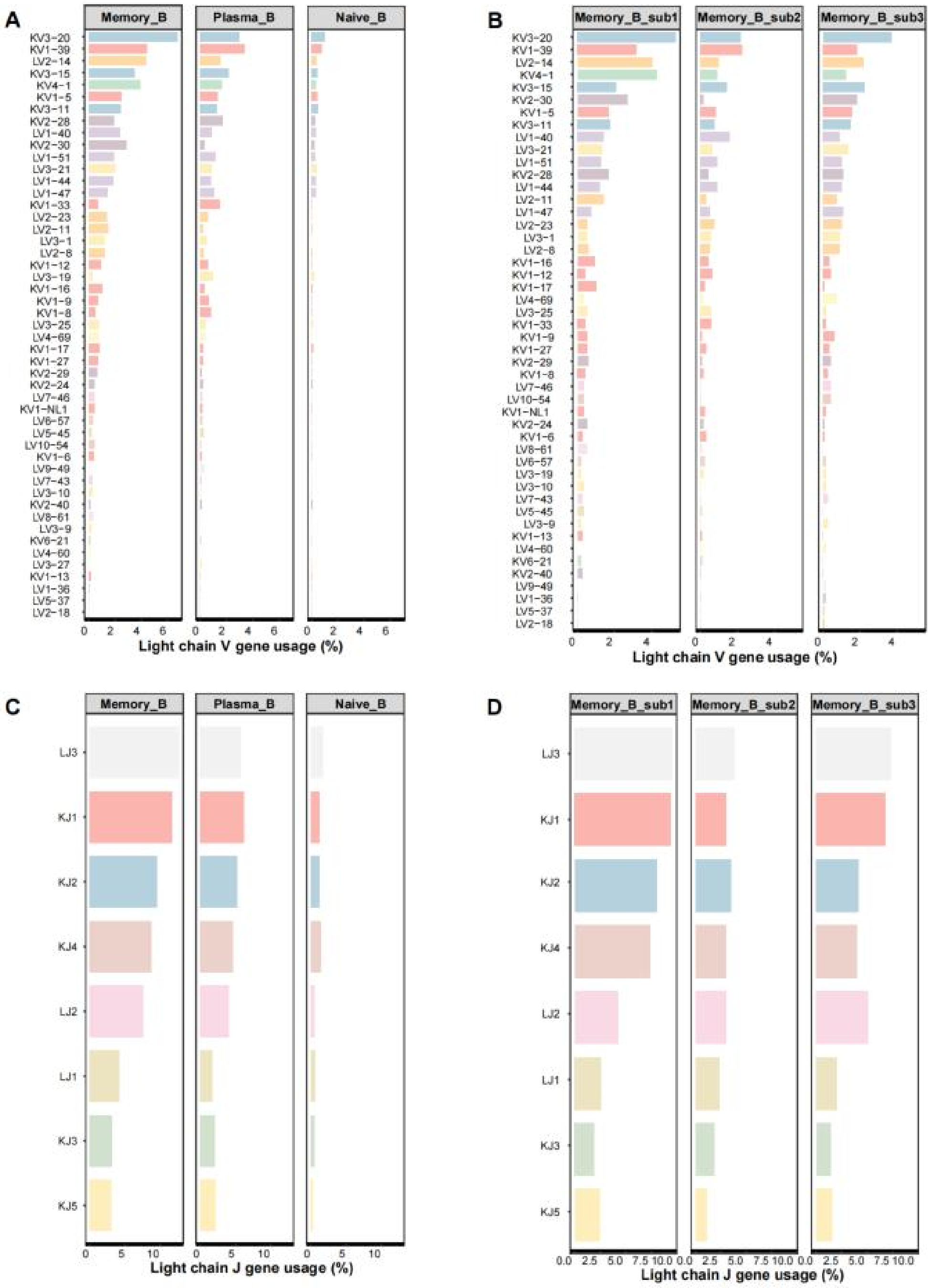

Supplemental Figure S2. The proportion of various V and J gene usage in Light chain among various B cells. A. V gene usage of light chains among major type of B cells. **B.** V gene usage of light chains among memory B cell subpopulations. **C.** J gene usage of light chains among major type of B cells. **D.** J gene usage of light chains among memory B cell subpopulations.

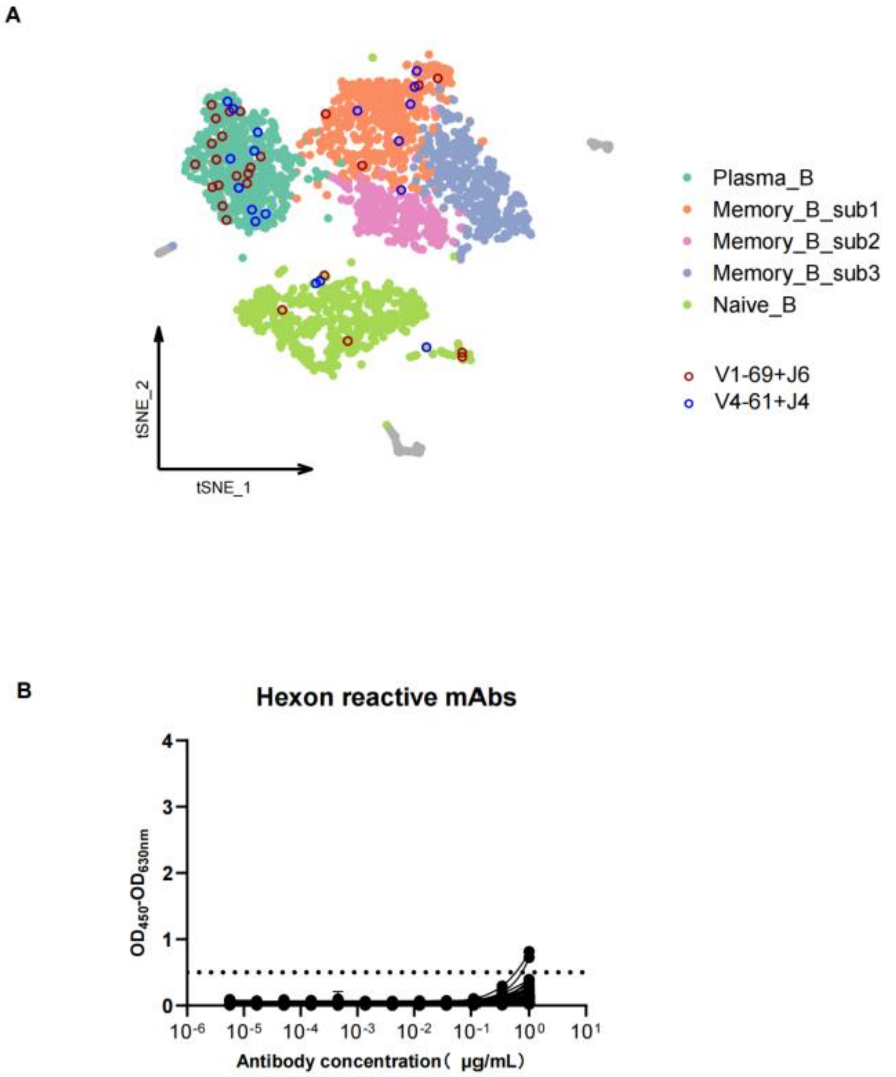

Supplemental Figure S3. Further antibody verification of specified V/J combination derived from other cell subpopulations. A. t-SNE visualization of the distribution of *V1-69/J6 and V4-61/J4* derived from other subpopulations. **B.** Detection of antibody binding activity to the HAdV-4 Hexon protein by ELISA.

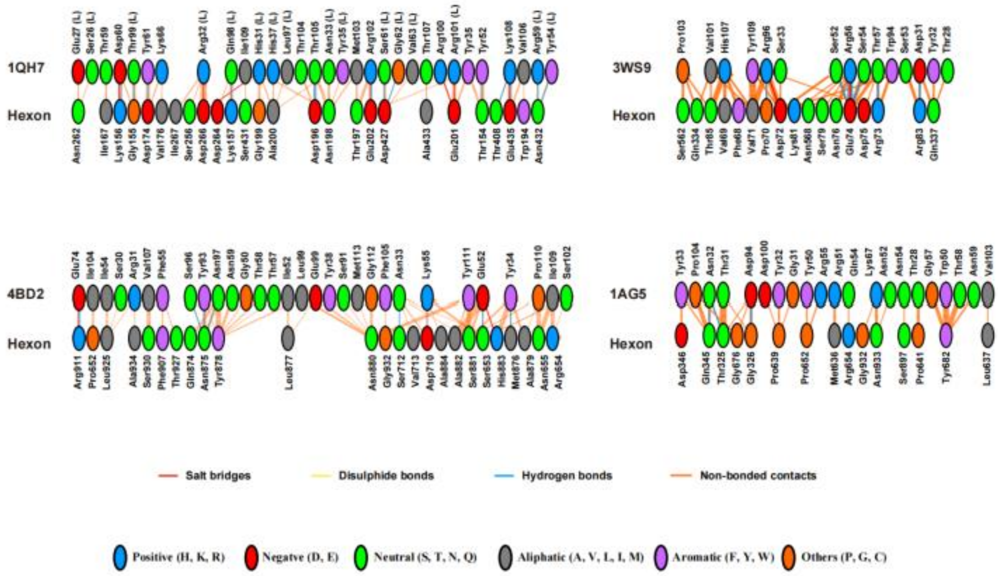

Supplementary Figure S4. Residue-residue interaction network at the interface between the Hexon domain and five monoclonal antibodies.

This figure presents the interaction networks at the binding interfaces of five Hexon–antibody complexes (1QH7, 3WS9, 4BD2, 1A5G, and the control 2CF4). For each complex, the participating Hexon residues and antibody residues are listed, with antibody light chain residues labeled with "L". Amino acid residues are represented by colored circles, with colors classified according to their physicochemical properties: positively charged (blue), negatively charged (red), neutral (green), hydrophobic (gray), aromatic (purple), and others (light blue). The lines connecting residues are colored according to the type of interaction: red for salt bridges, yellow for disulfide bonds, blue for hydrogen bonds, and orange for non- bonded contacts.

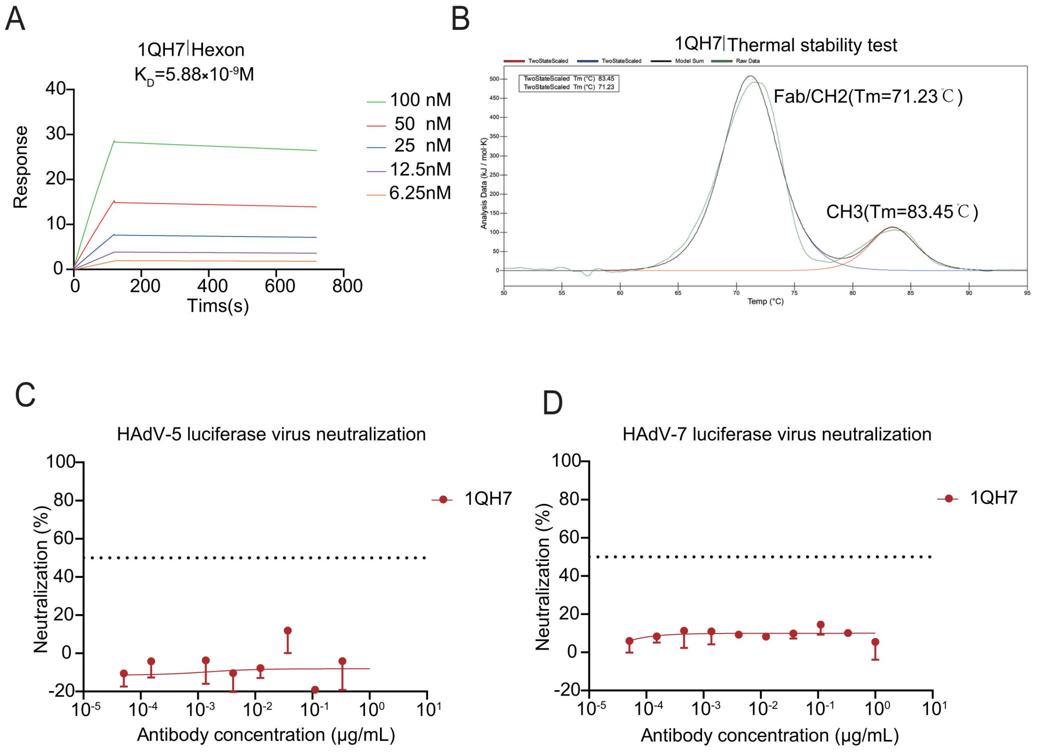

Supplemental Figure S5. Characterization of the Cross- Neutralization Activity, Binding Affinity, and Thermodynamic Properties of Monoclonal Antibody 1QH7. A. Neutralization of 1QH7 against Ad5-Luc virus in A549 cells. **B.** Neutralization of 1QH7 against Ad7-Luc virus in A549 cells. **C.** SPR sensorgrams and kinetics of 1QH7 binding to Hexon proteins. **D.** DSC measures the fundamental thermodynamic driving forces underlying the processes of protein stability, folding, and binding interactions for the 1QH7. Data in (A–D) are presented as the mean ± SD, derived from three technical replicates in a single representative experiment. The results were consistently validated across three independent biological replicates.

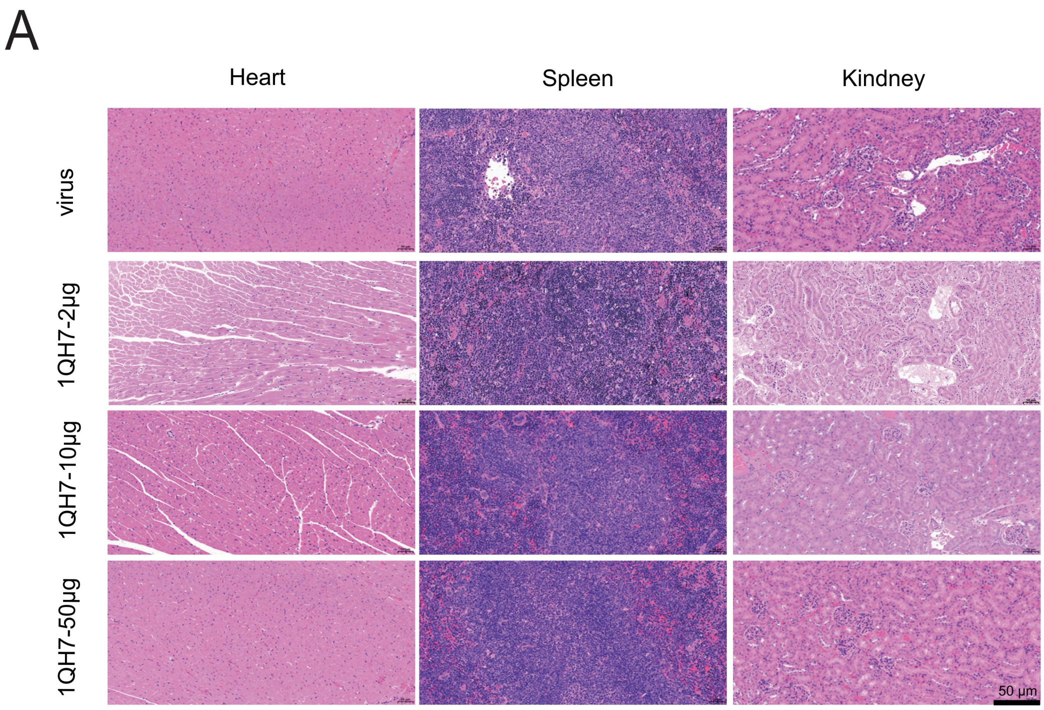

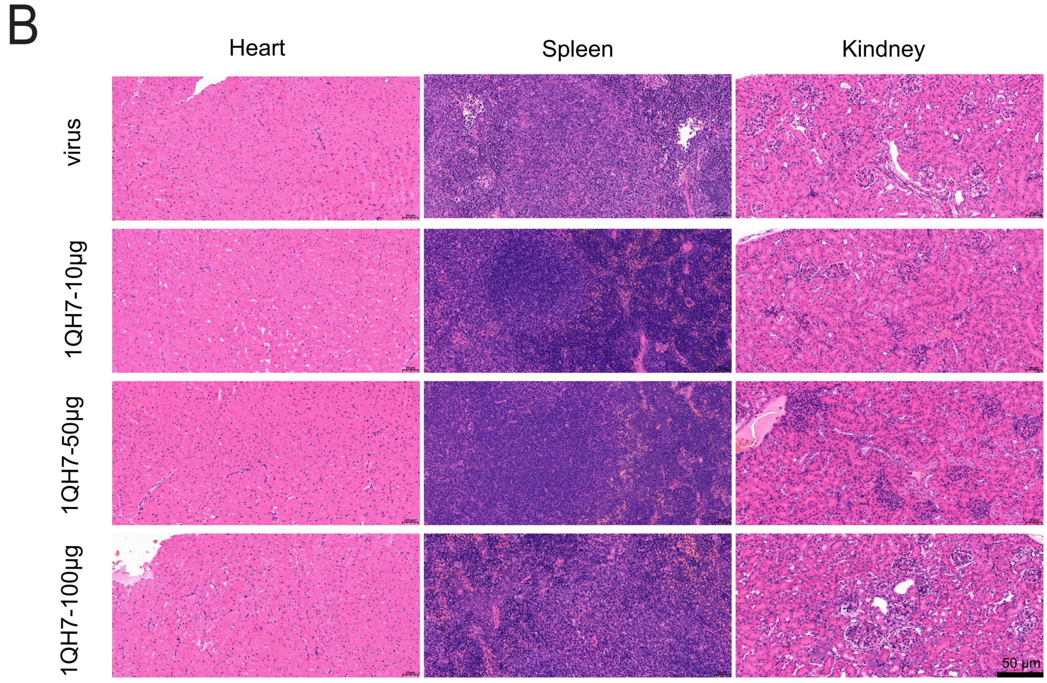

Supplemental Figure S6. Pathologic sections of the heart, spleen and kidneys three days after the attack of *Stat1*$^{-/-}$. **A.** Histopathological changes in the heart, spleen and kidneys of HAdV-4 infected mice collected at day 3 in the prophylactic groups. **B.** Histopathological changes in the heart, spleen and kidneys of HAdV-4 infected mice collected at day 3 in treatment group.

Supplemental Table S1. The number of various Ig among various cell population

Sub-group	Ig (n,%)					
	IgA	IgD	IgG	IgM	NA	Total
Plasma_B	2(0.32)	4(0.63)	4(0.63)	616(97.62)	5(0.79)	631(24.33)
Memory_B_sub1	104(18.41)	14(2.48)	98(17.35)	328(58.05)	21(3.72)	565(21.78)
Memory_B_sub2	189(21.25)	19(3.48)	132(60.63)	47(3.83)	37(10.80)	287(11.06)
Memory_B_sub3	61(44.58)	10(4.48)	174(31.13)	11(11.08)	31(8.73)	424(16.35)
Naive_B	57(8.30)	12(1.75)	29(4.22)	49(7.13)	540(78.60)	687(26.48)
Total	413(15.92)	59(2.27)	437(16.85)	1051(40.52)	634(24.44)	2594(100)

Supplemental Table S2. Characteristics of validated antibody germline gene combinations in memory B cells

V-J of Heavy chain	Memory_B_sub1			Memory_B_sub2			Memory_B_sub3			Name of Potent antibody
	Count (Number)	CDR3Length (mean)	SHM (mean)	Count (Number)	CDR3Length (mean)	SHM (mean)	Count (Number)	CDR3Length (mean)	SHM (mean)	
V1-18+J6	3	20.33	0.03	5	21.00	0.05	1	12.00	0.05	
V1-2+J4	11	16.73	0.04	6	17.33	0.06	19	15.42	0.08	1AG5; 2BT9
V1-69+J4	3	14.00	0.03	5	17.00	0.06	2	16.00	0.11	
V1-69+J6	4	21.75	0.02	7	19.29	0.06	6	19.50	0.06	4BD2
V2-5+J4	5	17.40	0.05	6	18.83	0.06	4	15.75	0.04	
V3-11+J6	1	13.00	0.02	1	19.00	0.05	2	13.00	0.06	3WS9
V3-48+J3	3	16.67	0.04	3	18.00	0.06	2	11.50	0.08	
V3-7+J3	4	19.50	0.03				4	15.50	0.10	
V4-34+J4	5	16.00	0.05	3	15.00	0.08	6	15.67	0.09	
V4-4+J5	2	14.00	0.04	3	18.33	0.07	2	17.50	0.08	
V4-61+J4	6	16.17	0.02				5	16.60	0.06	1QH7
Sum	47			**39**			**53**			

*Bold text indicates the antibody for the first-round validation. The number of total was 92.

Supplemental Table S3. Quality assessment of the antigen-antibody complex structures predicted by AlphaFold3.

Name	iptm	pTM	ranking_score	Ramachandran Plot (%)
1QH7	0.25	0.76	0.37	90.6
1AG5	0.23	0.76	0.35	90.2
3WS9	0.22	0.75	0.34	90.3
4BD2	0.23	0.75	0.35	89.6

Supplementary Table S4. Interface interaction analysis of the antigen-antibody complexes based on PISA.

Name	interface area (Å2)	NHB	NSB	NDS	CSS
1QH7	1448.3	19	16	0	0
1AG5	1092.2	5	0	0	0
3WS9	798.5	18	3	0	0
4BD2	1427.7	16	3	0	0
